# Nitrilases NIT1/2/3 Positively Regulate Flowering by Inhibiting *MAF4* Expression in Arabidopsis

**DOI:** 10.3389/fpls.2022.889460

**Published:** 2022-05-17

**Authors:** Shuang Yang, Tianqi Zhang, Ze Wang, Xiaofei Zhao, Rui Li, Jing Li

**Affiliations:** Department of Biological Sciences, College of Life Sciences, Northeast Agricultural University, Harbin, China

**Keywords:** *Arabidopsis thaliana*, nitrilase, auxin, *MAF4*, flowering time, chromatin modification

## Abstract

Three of the nitrilases (NITs), NIT1, NIT2, and NIT3, are ubiquitously existing in plant kingdom, which catalyze indole-3-acetonitrile into the most important auxin indole-3-acetic acid. Auxin is an indispensable hormone, which plays the important roles in almost all processes of plant growth and development. However, there are few reports on the regulation of flowering-time mediated by auxin. Here, we found that in Arabidopsis, *nit1/2/3* showed a late flowering phenotype in short days. To explore the molecular mechanism by which NIT1/2/3 regulate flowering time, we performed transcriptome sequencing of *nit1/2/3*. The results showed that the expression of a MADS-box transcription factor gene *MADS AFFECTING FLOWERING4* (*MAF4*) was dramatically increased in *nit1/2/3* comparing to wild type (WT). MAF4 is one of the paralogs of the potent flowering inhibitor FLOWERING LOCUS C (FLC). There are four other paralogs in FLC clade in Arabidopsis, including FLOWERING LOCUS M (FLM/MAF1), MAF2, MAF3, and MAF5. The late flowering phenotype of *nit1/2/3* could not be observed in the *maf4* background, indicating that the phenotype was specifically dependent on MAF4 rather than other FLC clade members. Interestingly, the expression of a lncRNA gene *MAS*, which is transcribed in the opposite direction of *MAF4*, was found significantly increased in *nit1/2/3*. Also, *MAS* has been reported to activate *MAF4* transcription by promoting histone 3 lysine 4 trimethylation (H3K4me3). As expected, H3K4me3 deposition at *MAF4* locus in *nit1/2/3* was highly enriched and significantly higher than that of WT. In summary, we show that NITs, NIT1/2/3, positively regulate flowering by repressing *MAF4* through manipulating H3K4me3 modification. Further study needs to be performed to explore the largely unknown mechanisms behind it.

## Introduction

Nitrilases (NITs), the ubiquitous enzymes in plant kingdom, catalyze the hydrolysis of organic cyanide into ammonia and corresponding carboxylic acids (Janowitz et al., 2009). Arabidopsis possesses four NITs, NIT1, NIT2, NIT3, and NIT4. The most primitive in evolution is NIT4, which is found in all plant species. It is capable of converting ß-cyanoalanine and functions in the process of cyanide detoxification. Quite similar to each other are NIT1/2/3 but less similar to NIT4 and are not active on ß-cyanoalanine. Also, NIT1/2/3 accept indole-3-acetonitrile (IAN) and convert it to the most important auxin, indole-3-acetic acid (IAA) (Bartling et al., 1992; Bartel and Fink, 1994; Schmidt et al., 1996; Dohmoto et al., 2000).

Through several pathways, IAA is produced from tryptophan (Trp), among which two pathways have been well-defined. One is IPA (Indole-3-pyruvic acid) pathway, which is considered as the predominant auxin biosynthesis pathway in plants (Mashiguchi et al., 2011) and the other is indole-3-acetaldoxime (IAOx) pathway, which may be restricted to Brassicaceae. In the IAOx pathway, Trp is converted to IAOx by CYP79B2 and CYP79B3 (Hull et al., 2000; Zhao et al., 2002). Then IAN is biosynthesized from IAOx by CYP71A13 (Kumari et al., 2015). Finally, NIT1/2/3 catalyze IAN to IAA. Also, IAOx is an important metabolic branch point, which is not only a precursor of IAA but also can be catalyzed to form camalexin and glucosinolates—two important biotic defense compounds. In addition to being metabolized from IAOx, IAN can be also produced from the degradation of indole glucosinolates by myrosinases (Halkier and Gershenzon, 2006; Burow et al., 2009; Kissen and Bones, 2009). It has been reported that IAN may not be the only substrate for nitrilases (Ishikawa et al., 2007; Agerbirk et al., 2008). Exogenous application of benzyl cyanide can lead to auxin-overproducer phenotype, due to the nitrilases-mediated conversion to phenylacetic acid (PAA), another natural auxin (Urbancsok et al., 2018). All in all, it has been confirmed that NIT1/2/3 are capable of catalyzing the biosynthesis of auxin.

The NIT1/2/3-mediated auxin biosynthesis has been reported to play roles in particular physiological situations or some stress conditions. For example, NIT1/2/3 are involved in promotion of hypocotyl elongation in response to high temperature (van der Woude et al., 2021). The NIT3-mediated IAA production regulates root development during sulfate deprivation (Kutz et al., 2002). The NIT1/2/3 promote symptom development and infection rate caused by *Plasmodiophora brassicae* (Grsic-Rausch et al., 2000). However, the role of NIT1/2/3 under normal growing conditions remains largely unknown.

The transition from vegetative to reproductive growth is a crucial switch in the life cycle of plants. In Arabidopsis, several signaling pathways have been found to synergically regulate flowering, including gibberellins (GAs), autonomous, vernalization, photoperiod, and age pathways (Leijten et al., 2018). These pathways eventually converge on the floral integration factors including FLOWERING LOCUS T (FT), SUPPRESSOR OF OVEREXPRESSION OF CO1 (SOC1), LEAFY (LFY) and APETALA1 (AP1), etc. to further manipulate gene expression in the meristem and lead to flowering (Fornara et al., 2010). The vernalization pathway is mediated to a large extent by the MADS-box transcription factor FLOWERING LOCUS C (FLC), and FLC is a potent repressor of flowering (Michaels and Amasino, 1999). Besides FLC, there are five FLC paralogs in Arabidopsis, including FLOWERING LOCUS M (FLM/MAF1) and MADS AFFECTING FLOWERING2-5 (MAF2-5). Like FLC, these MAF proteins are reported to act as flora repressors too (Ratcliffe et al., 2003; Gu et al., 2013). It has been suggested that FLC and the MAFs associate with another MADS-box domain protein SHORT VEGETATIVE PHASE (SVP) to directly repress the expression of *FT* and *SOC1*, resulting in flowering repression (Helliwell et al., 2006; Gu et al., 2013).

The multiple plant hormones including GAs, brassinosteroids (BRs), cytokinins (CKs), salicylic acid (SA), abscisic acid (ABA), jasmonates (JAs), and ethylene (ET) have been proved to regulate flowering-time (Davis, 2009). However, as a key hormone-regulating plant growth and development, auxin has been rarely reported to be involved in flowering-time control. In this study, we show that NIT1/2/3, the nitrilases catalyzing auxin biosynthesis are required for flowering in short days. Nitrilases NIT1/2/3 positively regulate flowering by repressing *MAF4* transcription through decreasing chromatin modification histone 3 lysine 4 trimethylation (H3K4me3).

## Materials and Methods

### Plant Material and Growth Conditions

The *Arabidopsis thaliana* Columbia-0 (Col-0) ecotype was used in this study. The mutants *nit1* (Salk_114153), *nit2* (Salk_207800), *nit3* (CS324250), and *maf4* (SALK_028506C) were obtained from the Arabidopsis Biological Resource Centre (ABRC, https://abrc.osu.edu/). Seeds were vernalized for 3 days at 4°C and grown in the soil. For seedlings cultured in medium, seeds were surface sterilized with 20% NaClO solution for 5 min, and subsequently washed with sterile water and plated on 1/2 MS medium with 3% sucrose, pH 5.8. The plants were grown, respectively, under long-day (16-h light/8-h dark) and short-day (8-h light/16-h dark) photoperiod with light at 100 μ mol·m^−2^·s^−1^, at 23°C and 60% relative humidity.

### Generation of Vectors and Transgenic Plants

Total RNA was isolated with TRIzol reagent and first-strand cDNA was synthesized using ReverTra Ace qPCR RT Kit (TOYOBO). The coding sequences of *NIT1*/*2*/*3* were amplified with TaKaRa Ex Taq (Stratagene) from cDNA using the primers NIT1/2/3-F and NIT1/2/3-R. To construct the vectors *35S::NIT1, 35S::NIT2*, and *35S::NIT3*, the obtained PCR products were then cloned into pCAMBIA2300 using the USER cloning method as previously described (Nour-Eldin et al., 2006).

To determine the expression patterns of the *NIT1*/*2*/*3*, the vectors *ProNIT1::GUS, ProNIT2::GUS*, and *ProNIT3::GUS*, expressing *GUS* driven by *NIT1*/*2*/*3* promoters, were generated. Then, 2-kb DNA fragments containing the putative *NIT1*/*2*/*3* promoters were amplified from genomic DNA with primers NIT1p/2p/3p-F and NIT1p/2p/3p-R. The PCR products were then cloned into the vector pCAMBIA3300 using the USER cloning method (Nour-Eldin et al., 2006).

Transgenic Arabidopsis were generated through *Agrobacterium tumefaciens*-mediated transformation (Zhang et al., 2006). Transformants *35S::NIT1, 35S::NIT2*, and *35S::NIT3* were selected by Kanamycin on 1/2MS medium. Transformants *ProNIT1::GUS, ProNIT2::GUS*, and *ProNIT3::GUS* were selected by Basta in the soil. The T3 homologous transgenic plants were used in the experiments. All primer sequences were listed in Supplementary Table 1.

### Arabidopsis Hybridization

To generate *maf4 nit1/2/3* double mutants, *nit1/2/3* were, respectively, hybridized with *maf4*. The flower buds prior to pollen maturation were emasculated in *maf4*, the stamens of *nit1/2/3* were used as pollen donors for hybridization. Bag the flower buds after cross-pollination, waiting for the seeds to be harvested. The harvested seeds were planted in the soil and the seedlings were used for genotyping with primers maf4-L, maf4-R, nit1/2/3-L, nit1/2/3-R, and LBb1.3. The homozygous hybrid plants were obtained for subsequent experiments.

To determine auxin distribution in *nit1/2/3*, transgenic plants *ProDR5::GUS* (provided by professor Sixue Chen of the University of Florida) was hybridized with *nit1*/*2*/*3*, respectively. Flower buds prior to pollen maturation were emasculated in *nit1*/*2*/*3*, the stamens of *ProDR5::GUS* were used as pollen donors. Bag the flower buds after cross-pollination, waiting for seeds to be harvested. The harvested seeds were planted in the soil and the seedlings were used for GUS staining. The seedlings with successful staining were used for genotyping with primers nit1/2/3-L, nit1/2/3-R, and LBb1.3. The homozygous hybrid plants were obtained for auxin distribution analysis. All primer sequences were listed in Supplementary Table 1.

### Determination of Flowering Time

The plants were grown in incubators at 23°C under long-day and short-day conditions, respectively. Record the date when the inflorescence is 0.5-cm tall. Count the number of rosette leaves on that day. At least 20 plants per genotype were counted and averaged for statistical analysis of results.

### Quantitative Real-Time PCR Analyses

Total RNA was isolated from 2-week old seedlings grown in short days using Ultrapure RNA Kit (Cwbio) and treated with TURBO DNA-free™ Kit (Thermo Fisher) to eliminate contaminated genomic DNA. The cDNA was synthesized using ReverTra Ace qPCR RT Kit (TOYOBO). A quantitative real-time PCR (qRT–PCR) was performed in triplicates each on three independently collected samples using Unique AptamerTM qPCR SYBR Green Master Mix (Nonogene). The expression of *ACTIN2* was used as an internal control. The data were calculated using the 2^−Δ*ΔCT*^ method (Livak and Schmittgen, 2001). The genes and primers used for qRT–PCR analyses were listed in Supplementary Table 1.

### The RNA Sequencing

Two-week old seedlings of wild type (WT) and *nit1*/*2*/*3* growing under short-day conditions were used for RNA sequencing (RNA-Seq). Three independent biological replicates of each genotype were conducted. Total RNA was extracted using Ultrapure RNA Kit (Cwbio). The construction of cDNA library and sequencing were performed by Berry Genomics Co. Ltd (Beijing, China) using Illumina NovaSeq6000 sequencing platform. Arabidopsis TAIR10 was used as the reference genome. Differentially expressed gene (DEG) analysis was conducted using DESeq2 with the criteria of absolute value of log_2_(foldchange) ≥1 and *p* < 0.05. The DEGs were visually enriched by Mapman (Thimm et al., 2004). The Kyoto Encyclopedia of Genes and Genomes (KEGG) database was used for pathway enrichment, significant enrichment pathways in DEGs were analyzed.

### The GUS Staining

The plants were infiltrated in GUS-staining buffer [0.05M NaPO_4_ (pH = 7.2), 10-mM EDTA, 0.1% TritonX-100, 2-mM K_3_Fe (CN)_6_, 2-mM K_4_Fe (CN)_6_, 2-mM X-Gluc], and incubated at 37°C for 8–12 h, followed by destaining in 75% ethanol. Poured out the 75% ethanol and added new 75% ethanol every 10 min. Repeated it for 4–5 times until the tissue was bleached. Samples were subsequently observed under light microscope and photographed.

### The ChIP qPCR

Two-week old seedlings (about 1.5 g) were submerged in 30 ml of isolation buffer A (10-mM Tris pH = 8.0, 400-mM sucrose, 10-mM Na–butyrate, 1% formaldehyde, 0.1-mM PMSF, 5-mM ß-Mercaptoethanol). Vacuum infiltrated for 10 min in an exicator at room temperature. Added 2.5 ml 2 M glycine to quench the crosslinking reaction. Took out the plant material, washed with water, wipe dried and ground in liquid nitrogen to a fine, dry powder. The ground samples were suspended in 30 ml of isolation buffer B (10-mM Tris pH = 8.0, 400-mM sucrose, 10-mM Na–butyrate, 0.1-mM PMSF, 5-mM ß-Mercaptoethanol, protease inhibitor cocktail). Incubated for 15 min at 4°C with gentle shaking. Filtered the solution through four layers of Miracloth into a new 50-ml tube. Centrifuged the filtrate for 20 min at 2,880 g at 4°C. Resuspended the pellet in 1 ml isolation buffer C (10-mM Tris pH = 8.0, 250-mM sucrose, 10-mM Na–butyrate, 10-mM MgCl_2_, 1% TritonX-100, 0.1-mM PMSF, 5-mM ß-Mercaptoethanol, protease inhibitor cocktail). Centrifuged at 12,000 g for 10 min at 4°C. Resuspended the nuclei pellet in 300-μl isolation buffer D (10-mM Tris pH = 8.0, 1.7-M sucrose, 10-mM Na–butyrate, 2-mM MgCl_2_, 0.15% TritonX-100, 0.1-mM PMSF, 5-mM ß-Mercaptoethanol, protease inhibitor cocktail). Added 1,500-μl of isolation buffer D to a tube and overlayed this layer with the nuclei suspension and centrifuged for 1 h at 16,000 g at 4°C. Resuspended the pellet in 320-μl nuclei lysis buffer (50-mM Tris pH = 8.0, 10-mM EDTA, 0.4% SDS, 0.1-mM PMSF, protease inhibitor cocktail). Then, DNA was fragmented by sonicating for 20 min. Immunoprecipitation (IP), IP wash, elution, crosslink reversal, and DNA cleanup were performed with One-Day Chromatin Immunoprecipitation Kits (17-10086, Millipore) according to the manufacturer's instruction. The fragmented-DNA was incubated with rabbit polyclonal anti-H3K4me3 (04-745, Millipore, 1:250) at 4°C for 6 h with rotation. The qPCR was performed using Unique Aptamer TM qPCR SYBR Green Master Mix (Nonogene). Relative enrichment of H3K4me3 in each DNA region was normalized to input DNA. Then, ΔCt [normalized ChIP] = Ct [ChIP] – [Ct (Input) – log_2_(Input Dilution Factor)], % Input = 2^−Δ*Ct*^[normalized ChIP]). Primers used for ChIP qPCR were listed in Supplementary Table 1.

## Results

### Nitrilases NIT1/2/3 Were Required for Flowering Under Short-Day Conditions

**Figure 1:**
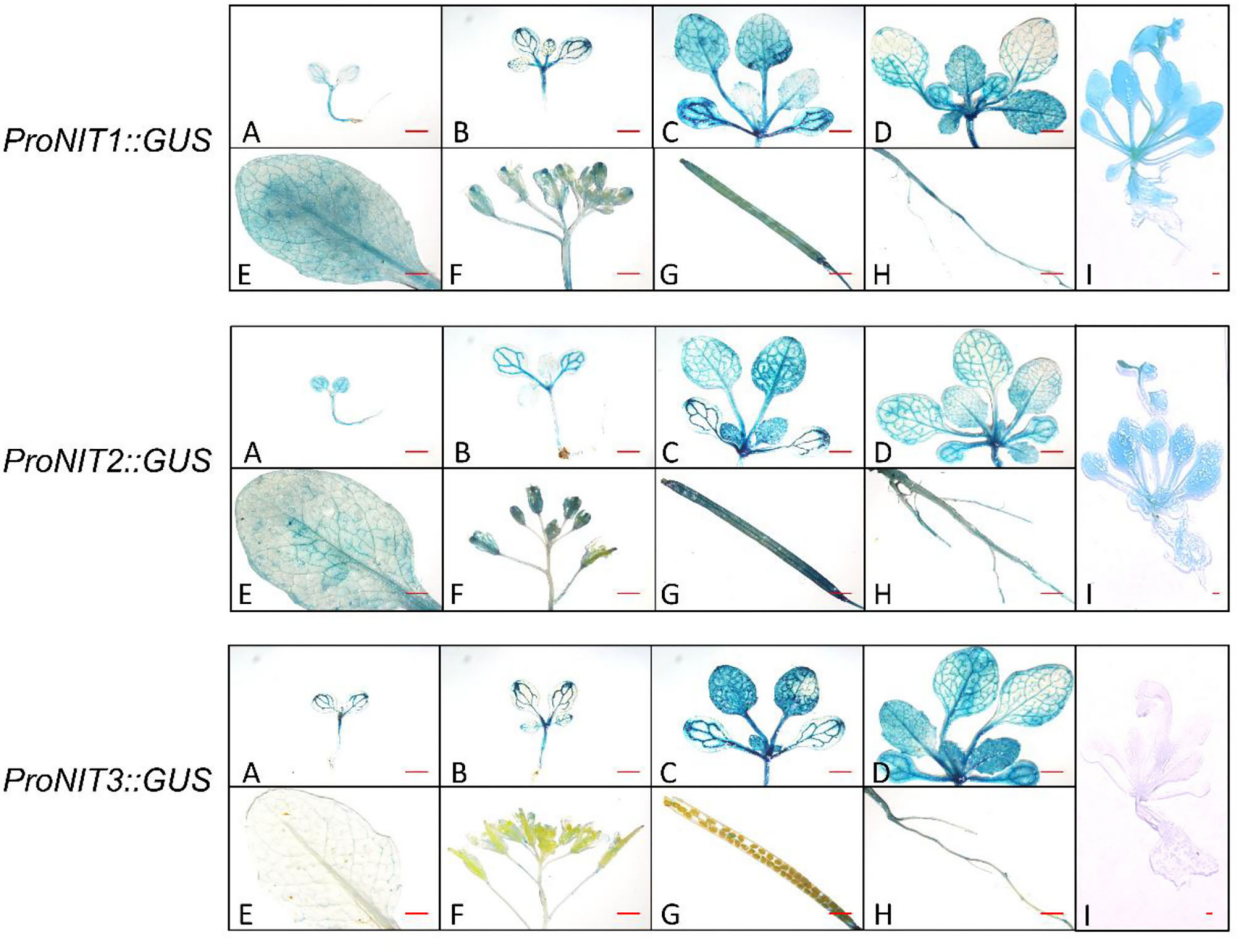
Analysis of *NIT1*/*2*/*3* promotor activity. Spatial expression pattern of *GUS* under control of *NIT1/2/3* promoter. For cotyledon **(A)**, two-leaf stage **(B)**, four-leaf stage **(C)**, six-leaf stage **(D)**, mature leaf **(E)**, inflorescence **(F)**, pod **(G)**, root **(H)**, and adult plant **(I)**, Bar = 1 mm.

The previous studies showed that NIT1/2/3 are mainly involved in pathogen defense, root growth, and development under stress conditions (Grsic-Rausch et al., 2000; Lehmann et al., 2017; van der Woude et al., 2021). As enzymes catalyzing the biosynthesis of plant hormone IAA, the function of NIT1/2/3 under normal conditions has been rarely reported. Using reporter gene *GUS*, we investigated the expression profile of *NIT1/2/3*. It was found that the *NIT1*/*2*/*3* were widely expressed in all stages of growth and development (except *NIT3*, its expression level decreased greatly and became undetectable after bolting) (Figure 1). Therefore, we speculated that NIT1/2/3 should play a role in normal growth and development, not just under adverse conditions.

**Figure 2:**
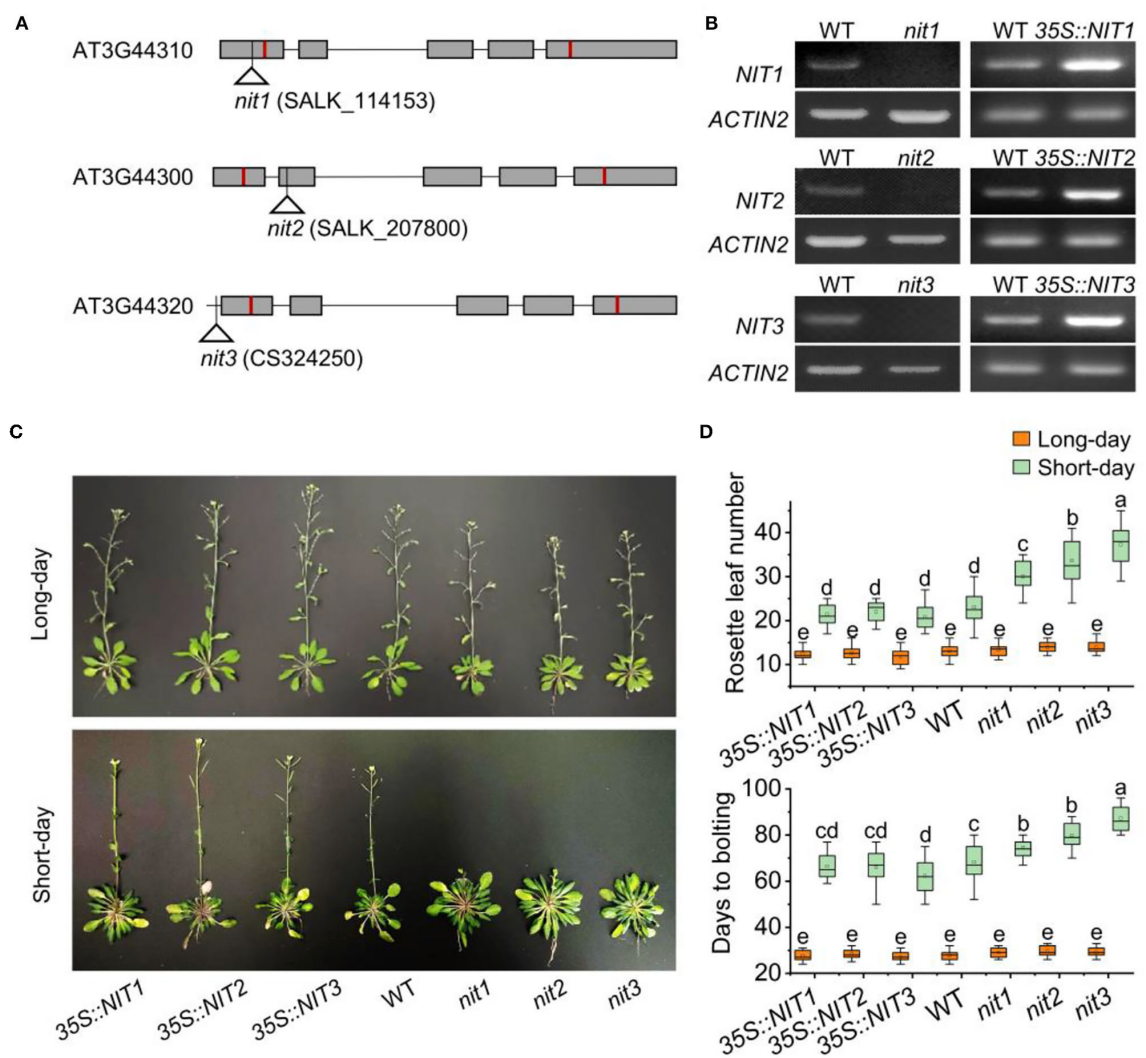
NIT1/2/3 regulate flowering under short day conditions. **(A)** The T-DNA insertion sites in *NIT1*/*2*/*3*. Squares and lines represent exons and introns, respectively. The red line shows the start and stop codons. The insertion sites are represented by a triangle. **(B)** Semiquantitative RT–PCR analysis of *NIT1/2/3* gene expression. **(C)** Flowering phenotypes of *35S::NIT1, 35S::NIT2, 35S::NIT3*, WT, *nit1, nit2*, and *nit3*. **(D)** Days to bolting and rosette leaf numbers in *35S::NIT1, 35S::NIT2, 35S::NIT3*, WT, *nit1, nit2*, and *nit3*. At least 20 plants of each genotype were used for statistical analysis of flowering time. Box plots display median (line), mean (block), interquartile range (box), and whiskers (extending 1.5 times the interquartile range). Significant differences are denoted with distinct letters (Tukey's *post hoc* test, *p* < 0.05).

To explore the function of NIT1/2/3, transgenic plants *35S::NIT1, 35S::NIT2*, and *35S::NIT3* were constructed, and T-DNA knock out mutants *nit1, nit2*, and *nit3* were obtained (Figures 2A,B and Supplementary Figure 1). Under long-day conditions, no significant difference in flowering-time was observed in *35S::NIT1, 35S::NIT2, 35S::NIT3, nit1, nit2*, and *nit3* comparing to WT. Consistently, the number of rosette leaves and the days to bolting in these mutants did not show any alteration. Under short-day conditions, transgenic plants overexpressing *NIT1/2/3* did not show any phenotype; however, flowering in *nit1, nit2*, and *nit3* were significantly postponed (Figure 2C). Comparing to WT, the bolting times were 7–15 days later, and the rosette leaf number increased by 7–14 in *nit1, nit2*, and *nit3* (Figure 2D).

**Figure 3:**
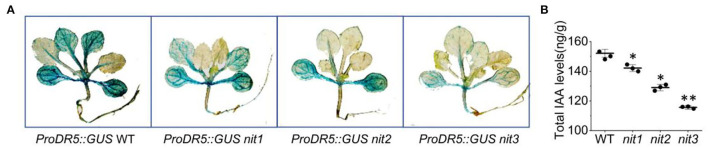
The IAA level and distribution in *nit1/2/3*. **(A)** IAA distribution in seedings of WT, *nit1, nit2*, and *nit3*. **(B)** The IAA levels in seedings of WT, *nit1, nit2*, and *nit3*. The 2-week-old seedlings were used for GUS staining and IAA level analysis. The experiments were performed in short days. The error bars represent the standard error (three biological repeats). The asterisks at the top of the bar indicate significant differences between *nit1/2/3* and WT, *p* < 0.05(*) or *p* < 0.01(**) by Student's *t*-test.

We further detected IAA content and distribution in *nit1/2/3*. As shown in Figure 3A, the expression of *GUS* driven by DR5 (a promoter responsive to auxin) in *nit1/2/3* was significantly weaker than that in WT. Consistently, the total IAA content in *nit1/2/3* was lower comparing to WT (Figure 3B). The IAA content and the auxin level presented by GUS signal were the lowest in *nit3* which flowered the latest. We speculated that the late flowering phenotype in *nit1/2/3* was related with auxin.

### Deficiency of *NIT1*/*2*/*3* Activated the Expression of *FLC* Clade Genes and Inhibited the Expression of Flowering Integration Factor Genes

Nitrilases NIT1/2/3 have been identified as enzymes that catalyze the biosynthesis of IAA. However, no IAA-mediated flowering pathway had been reported so far. To determine how the NIT1/2/3 affect flowering, we performed transcriptome sequencing in 2-week old seedlings of *nit1*/*2*/*3* and WT grown in short days. Comparing with WT, 1,208, 1,886, and 1,662 genes were differently expressed in *nit1, nit2*, and *nit3*, respectively. Among these differently expressed genes (DEGs), 645 genes were shared in *nit1*/*2*/*3*. The molecular functions and signal pathways enriched by GO and KEGG analysis overlapped greatly between the *nit1/2/3* (Supplementary Figure 2), indicating the functional similarity of the NIT1/2/3 proteins. Since it was difficult to determine the flowering pathways affected by NIT1/2/3 only through GO and KEGG analysis, we thus investigated the expression profile of the key genes involved in all the flowering pathways.

**Figure 4:**
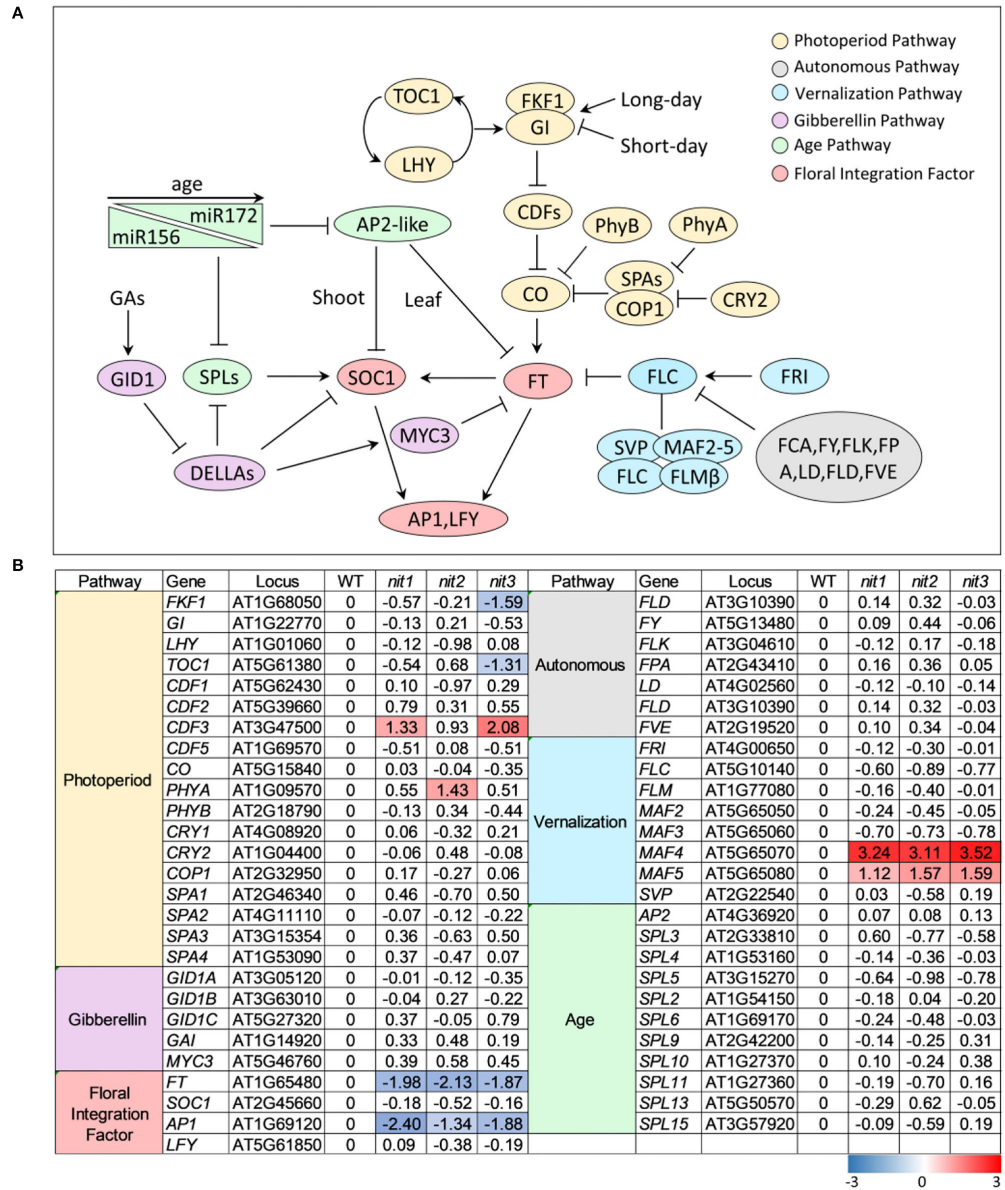
The transcription profile of key genes involved in different flowering pathways. **(A)** Outline of flowering pathways in Arabidopsis. **(B)** The transcription profile of key genes involved in different flowering pathways. The value is log_2_(Fold Change). The DEGs with log_2_(Fold Change) ≥1 and *p* ≤ 0.05 are highlighted in colors. Red refers to increased expression and blue refers to decreased expression.

In Arabidopsis, flowering is coordinately regulated by multiple pathways, including the pathways of photoperiod, GA, autonomous, vernalization, and age. These signaling pathways eventually converge on several floral integration factors (FT, SOC1) to activate downstream floral meristem genes (*LFY, AP1*) and trigger the transition from vegetative to reproductive phase (Figure 4A). As shown in Figure 4B, no genes in GA pathway, autonomous pathway, and age pathway were found to be differentially expressed in *nit1*/*2*/*3*. In the photoperiod pathway, *FKF1* (F BOX 1) and *TOC1* (TIMING OF CAB EXPRESSION 1) were repressed in *nit3, CDF3* (CYCLING DOF FACTOR 3) was promoted in *nit1* and *nit3*, and *PHYA* (PHYTOCHROME A) was promoted in *nit2*. The most notable DEGs were *MAF4* and *MAF5*, two paralogs of *FLC* in the vernalization pathway. The expression of both genes was significantly increased in *nit1*/*2*/*3*, especially *MAF4*, the expression of which increased 8–10 times. Consistent with the activation of flowering inhibitors, the expression levels of flowering integration factor genes *FT* and *AP1* decreased significantly in *nit1*/*2*/*3*.

**Figure 5:**
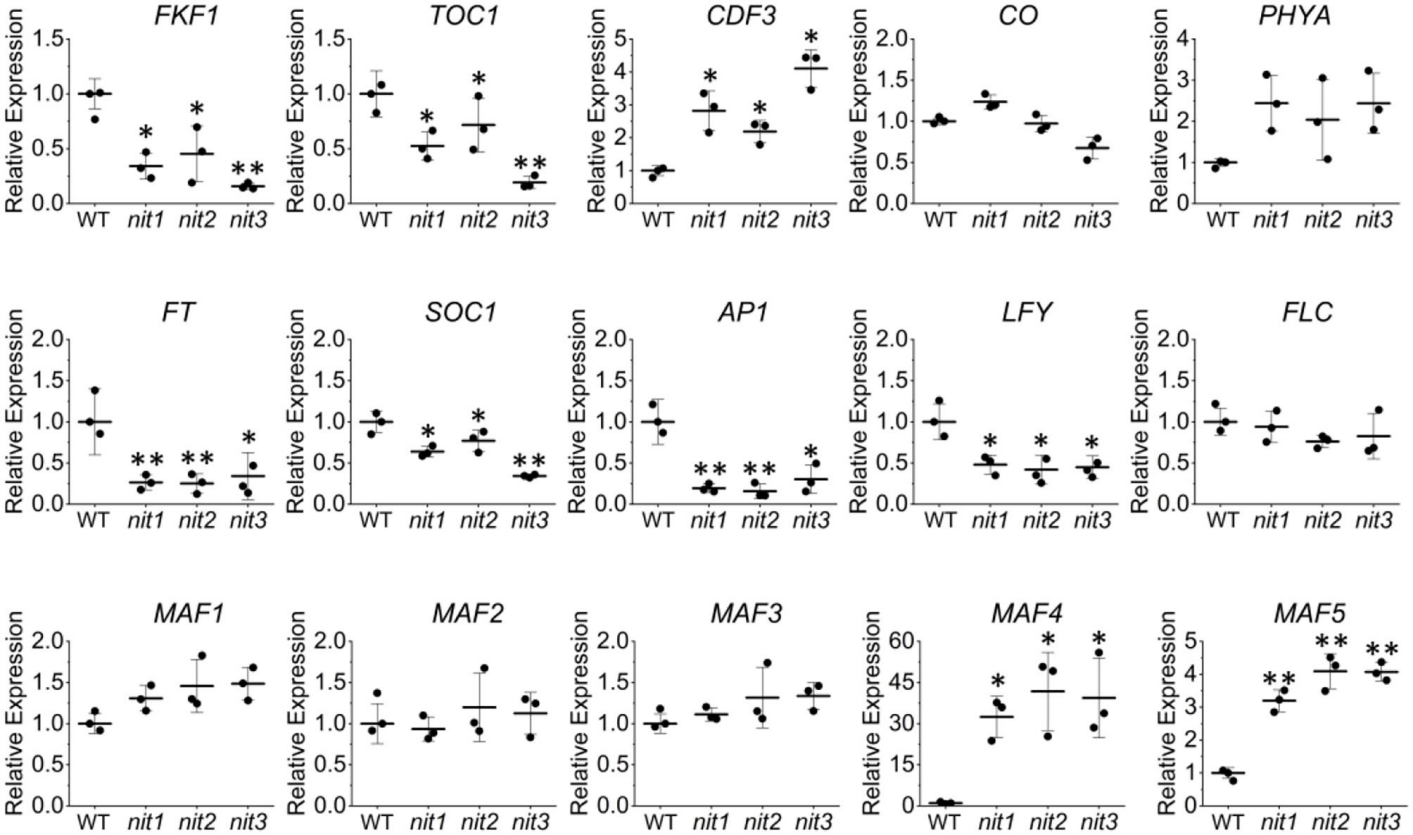
Quantitative real-time–PCR validation of the key genes' expression in the flowering pathways. The error bars represent the standard error (three biological repeats). The asterisks at the top of the bar indicate significant differences between *nit1/2/3* and WT, *p* < 0.05(*) or *p* < 0.01(**) by Student's *t*-test.

To confirm the transcriptome data, qRT–PCR analysis of the above DEGs and several other flowering related genes was performed. As shown in Figure 5, the expression alterations of the detected genes in *nit1*/*2*/*3* were largely consistent with the transcriptome results. Slightly different from the transcriptome data, qRT–PCR analysis showed that the expression of *MAF4* increased 30, 40, and 45 times respectively in *nit1, nit2*, and *nit3*, which was greater than that of transcriptome data. This may be due to the low expression level of *MAF4* in the WT. However, the conclusion based on the transcriptome and qRT–PCR analyses were consistent, that is, MAF4 and MAF5 or photoperiod pathway maybe involved in NIT1/2/3-deficiency-mediated flowering delay.

### Nitrilases NIT1/NIT2/NIT3-Regulated Flowering by Manipulating the Expression of *MAF4*

**Figure 6:**
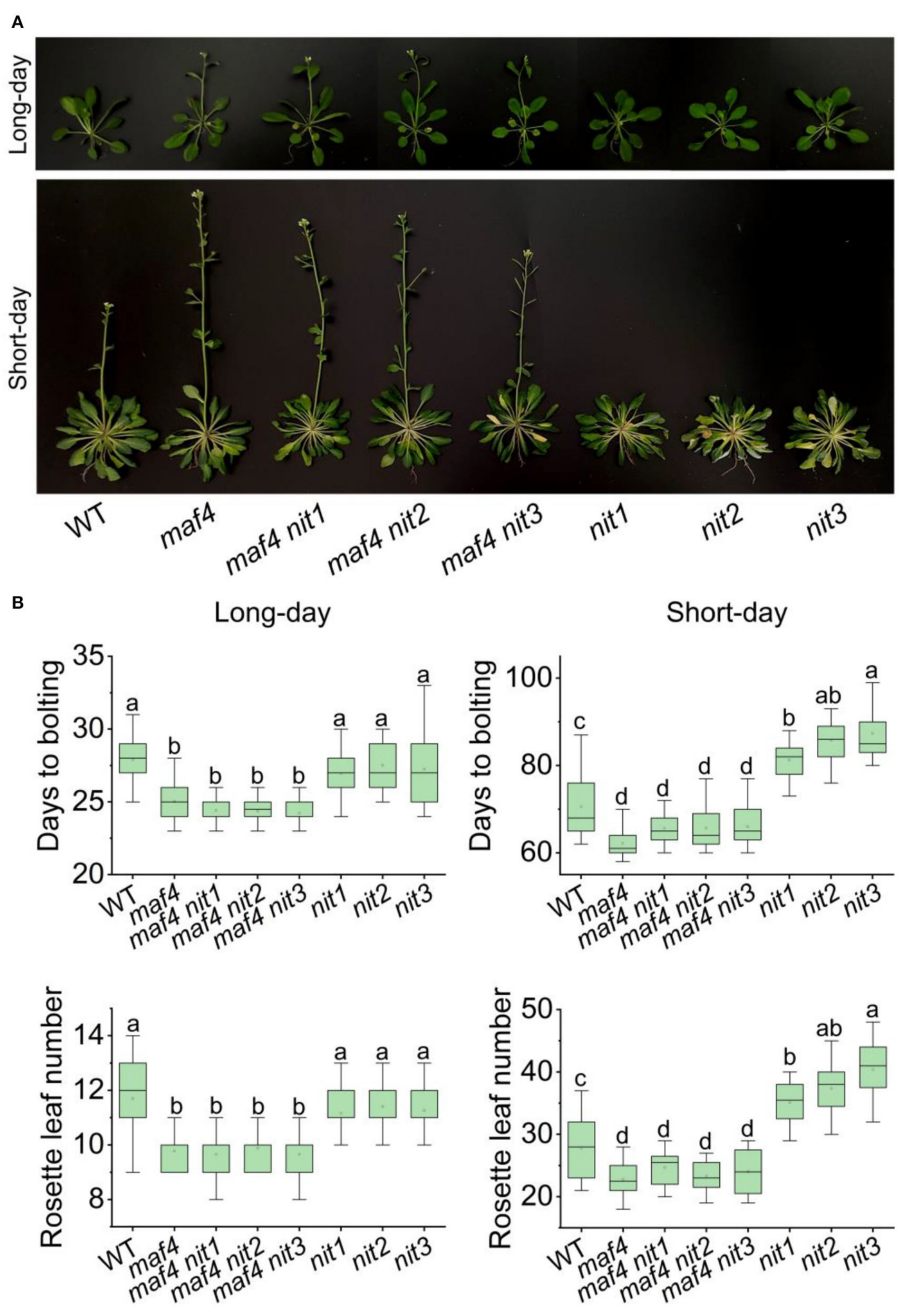
Flowering phenotype of *nit1*/*2*/*3* in the *maf4* background. **(A)** Flowering phenotypes of WT, *maf4, maf4 nit1, maf4 nit2, maf4 nit3, nit1, nit2*, and *nit3*. **(B)** Days to bolting and rosette leaf numbers in different mutants. At least 20 plants of each genotype were used for statistical analysis. Box plots display median (line), mean (block), interquartile range (box), and whiskers (extending 1.5 times the interquartile range). Significant differences are denoted with distinct letters (Tukey's *post hoc* test, *p* < 0.05).

In *nit1/2/3*, the expression level of *MAF4* altered the most comparing to that of the other DEGs in flowering pathways. Therefore, we speculated that MAF4 might play a major role in NIT1/2/3-deficiency-mediated flowering delay. In long days, *maf4* showed slightly (3–4 days) earlier flowering and no significant difference in flowering was observed in *maf4 nit1/2/3* comparing to *maf4* (Figure 6). Since NIT1/2/3 do not affect flowering in long days, the early flowering in *maf4* and *maf4 nit1/2/3* were caused by *MAF4* deficiency, indicating that MAF4 slightly inhibited flowering in long days. In short days *nit1/2/3* showed delayed flowering; however, the late flowering in *nit1/2/3* could not be observed in the *maf4* background (Figure 6) indicating that the phenotype was MAF4 dependent. While MAF5 and the photoperiod pathway have little effect.

### Effect of NIT1/2/3 on the Expression of Alternative Splice Variants of *MAF4*

**Figure 7:**
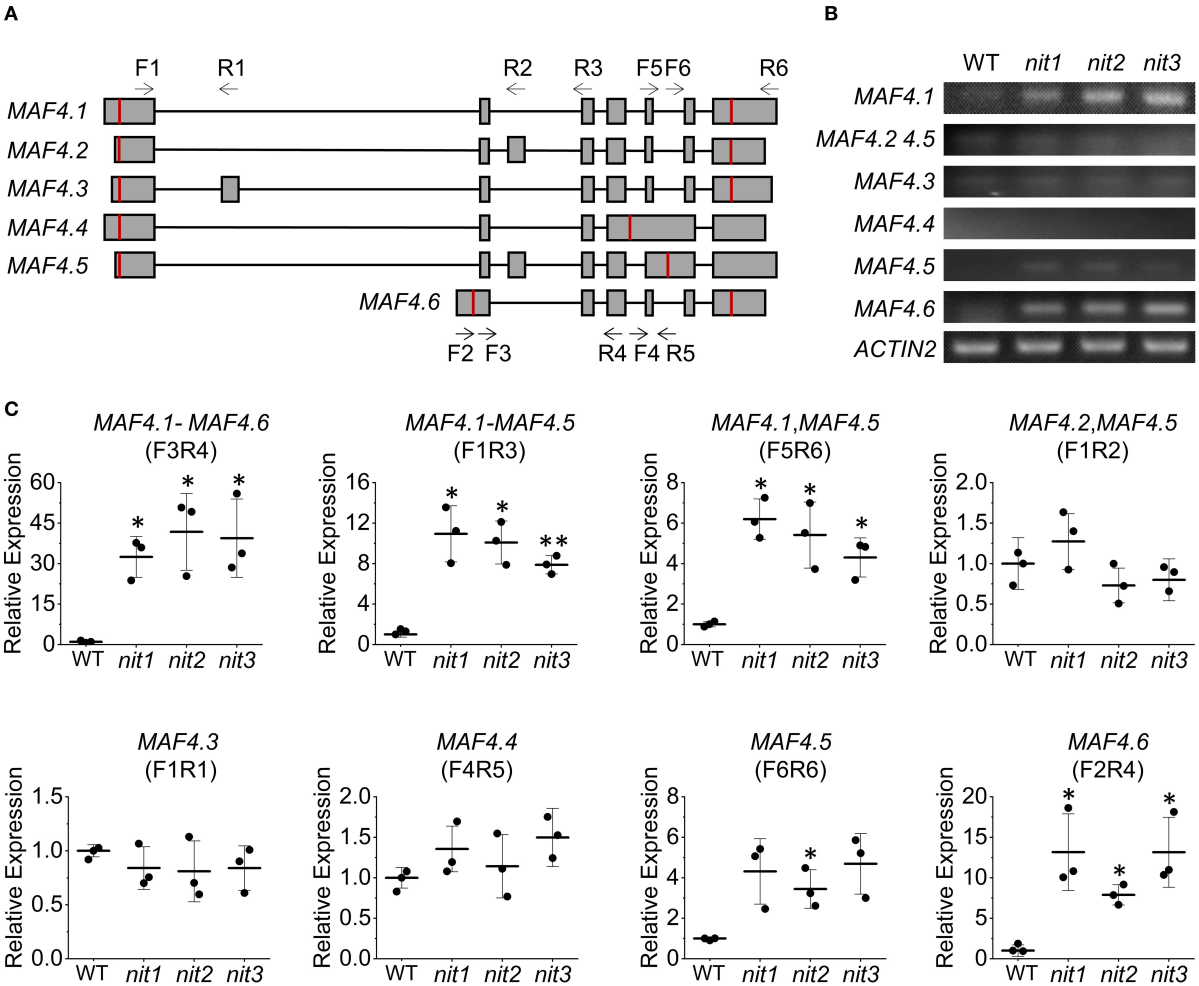
Relative expression levels of *MAF4* alternative splicing variants. **(A)** The alternative splicing of *MAF4*. Squares and lines represent exons and introns, respectively. The red line shows the start and stop codons; F refers to forward primer and R refers to reverse primer. Arrow indicates primer direction. **(B)** Semiquantitative RT–PCR analysis of *MAF4* splicing variants. **(C)** Relative expression of *MAF4* splicing variants. The error bars represent the standard error (three biological repeats). The asterisks at the top of the bar indicate significant differences between *nit1/2/3* and WT, *p* < 0.05(*) or *p* < 0.01(**) by Student's *t*-test.

The *FLC* clade members including *FLC* and *MAF1-5* all generate multiple alternative splice variants. The transcriptome and qRT–PCR results showed that the deficiency of *NIT1/2/3* activated the expression *MAF4* and *MAF5*; however, it was not known how the alternative splicing of each gene was regulated. We thus investigated expression of six alternative splice variants generated by *MAF4* (Figure 7A) in *nit1/2/3*. In the WT, the expression level of all six splice variants were quite low. While in *nit1/2/3* mutants, the transcript of *MAF4.1* and *MAF4.6* increased significantly, and the level of other splice variants did not alter significantly (Figures 7B,C). We further detected alternative splice variants' expression of other five *FLC* clade genes. The results showed that only the expression level of splice variants *MAF5.1* and *MAF5.2* increased in *nit1/2/3*. For the other *FLC* clade genes, no altered expression of splice variants was observed (Supplementary Figure 3).

### Nitrilases NIT1/2/3 Regulated *MAF4* Expression Through H3K4me3

**Figure 8:**
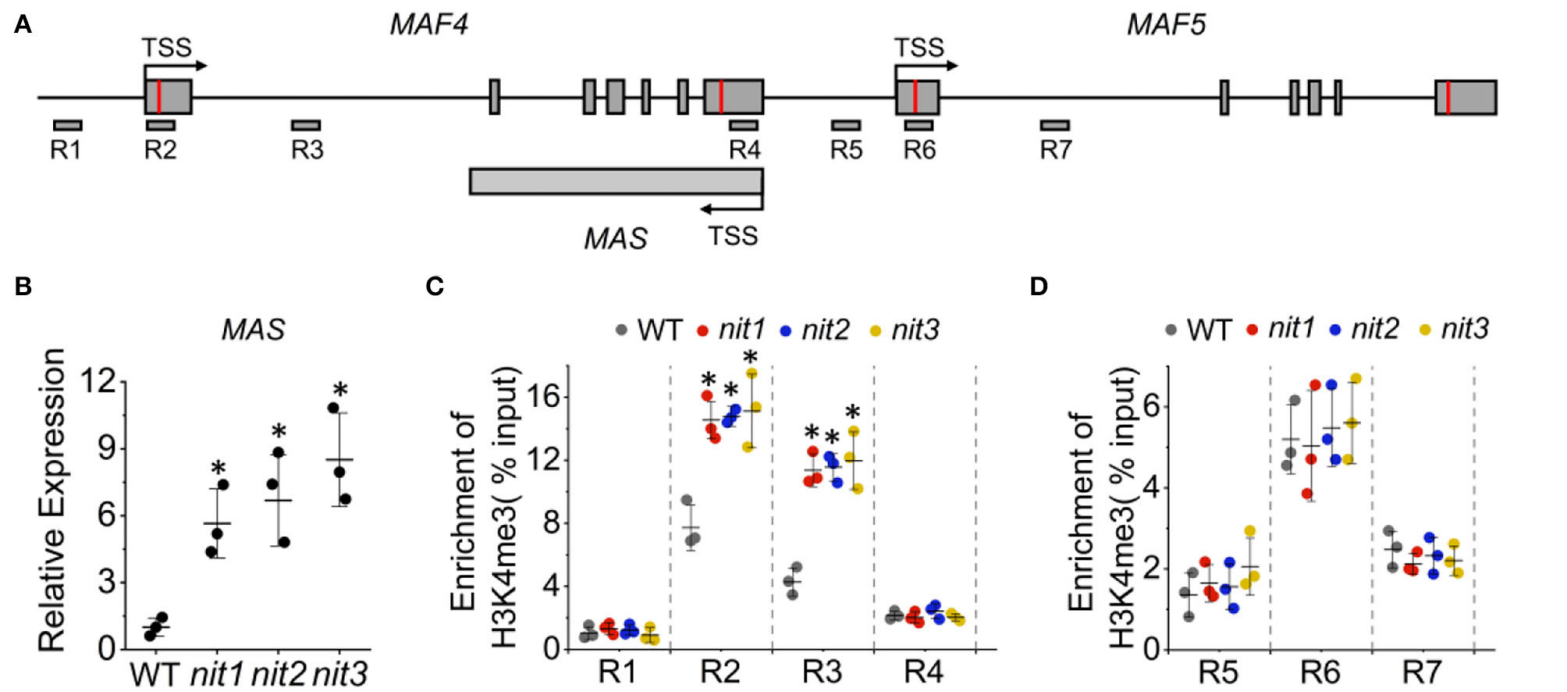
The *MAS*-mediated activation of *MAF4* gene expression. **(A)** The schematic representation of *MAF4, MAF5*, and *MAS* locus. The positions of primers (R1–R7) used for ChIP qPCR are indicated. **(B)** The expression of *MAS* in WT and *nit1*/*2*/*3* lines. **(C)** H3K4me3 levels at *MAF4* locus in WT and *nit1/2*/*3*. **(D)** H3K4me3 levels at *MAF5* locus in WT and *nit1/2*/*3*. Enrichment of H3K4me3 was determined by ChIP qPCR. The error bars represent the standard error (three biological repeats). The asterisks at the top of the bar indicate significant differences between *nit1/2/3* and WT, *p* < 0.05(*) by Student's t-test.

The expression of *FLC* and its paralogs *MAF*s are regulated mostly at the epigenetic level (He and Amasino, 2005; Alexandre and Hennig, 2008) and a substantial number of positive and negative regulators have been described (listed in Supplementary Table 2). To explore whether these regulators mediate the increase of *MAF4* and *MAF5* in *nit1/2/3*, we analyzed the expression of the known regulators of *FLC* and *MAF*s in *nit1/2/3*. Both transcriptome data and qRT–PCR analysis showed no significant alteration in the expression of these genes. Interestingly, the expression of a lncRNA gene *MAS* was found significantly increased in *nit1/2/3* under short-day conditions (Figures 8A,B). Also, *MAS* is a NAT–lncRNA, which is the natural antisense transcript (NAT) that is transcribed in the opposite direction of *MAF4* (Figure 8A). In Arabidopsis, H3K4me3 has been implicated in transcriptional activation of genes including *MAF4* (Gu et al., 2009; Liu et al., 2010). The lncRNA, *MAS*, activates *MAF4* by binding WDR5a, the core component of the COMPASS-like complexes, and guiding WDR5a to *MAF4* to promote H3K4me3 (Zhao et al., 2018). Since *MAF4* is adjacent to *MAF5* in genomic DNA, theoretically, *MAS* may also activate the expression of *MAF5* by the same mechanism. To explore whether NIT1/2/3 regulate the expression of *MAF4* and *MAF5* through *MAS*-mediated H3K4me3, we detected H3K4me3 levels at the *MAF4* and *MAF5* locus in WT and *nit1/2/3*. As shown in Figures 8C,D, in *nit1/2/3*, H3K4me3 deposition at the transcription start site (TSS) and the first intron of *MAF4* locus was highly enriched and significantly higher than that of WT. While the levels of H3K4me3 remained unaltered at the *MAF5* locus, these results suggested that *MAF4* was activated by H3K4me3 at its TSS and the first intron locus in *nit1/2/3*, and the H3K4me3 was possibly promoted by *MAS*. Our results also indicated that *MAS* was more likely to activate its sense overlapping gene *MAF4* than its adjacent gene *MAF5*.

## Discussion

The transition from vegetative to reproductive growth is a crucial switch in plants, which affects the survival of individuals and continuation of species. As such, the regulation of flowering-time must be tightly and precisely controlled. Plant hormone signaling is known to regulate almost all growth and development processes and is also indispensable in flowering-time control. The multiple plant hormones have been proved to regulate floral transition (Davis, 2009; Izawa, 2021). Among them, GA is the best understood one and an unquestioned flowering promoter (Davis, 2009; Bao et al., 2020). The other hormones including BRs, CKs, and SA are considered to be positive regulators of flowering and ABA, JA, and ET are considered to be negative regulators of flowering (Davis, 2009). Auxin is a key hormone in the control of plant growth and development and has been reported to regulate many processes in plant morphogenesis. However, reports on the relation between auxin and flowering have been very limited (Davis, 2009). In this study, we discovered that NIT1/2/3, the nitrilases catalyzing auxin biosynthesis, positively regulate flowering in short days by repressing transcription of the floral inhibitor *MAF4*. This suggested that either auxin-mediated signaling was involved in flowering-time control, or that other metabolites affected by NIT1/2/3-activity-regulated flowering through an auxin-independent pathways. Auxin controls stem cell fate determination and organ differentiation at shoot and root apical meristem (Wolters and Jürgens, 2009; Hata and Kyozuka, 2021). Furthermore, auxin moves through the phloem or *via* transport proteins, which makes it as an excellent candidate for the signal transportation (Robert and Friml, 2009). Thus, in theory, auxin could be a major signaling of phase change and have a floral-inductive role. However, further evidence is needed on whether NIT-mediated auxin synthesis is involved in flowering regulation.

The FLC and its five paralogs MAFs are negative regulators of flowering, among which FLC is considered to play a predominant role in repression of floral transition. In recent years, the function of MAFs in flowering-time control has been gradually discovered. The FLM/MAF1 and MAF2 appear to act in the induction of flowering by elevated temperature (Balasubramanian et al., 2006). The MAF2 prevents flowering caused by insufficient vernalization (Ratcliffe et al., 2003). The MAF3 participates in flowering-time regulation by affecting photoperiod pathway (Gu et al., 2013). In this study, we showed that NIT1/2/3 positively regulates flowering by inhibiting the expression of *MAF4*. Also, the effect of NIT1/2/3 specifically depends on MAF4, since the late flowering phenotype of *nit1/2/3* could not be observed in the *maf4* background. Though the expression level of *MAF5* increased in *nit1/2/3*, it had little effect on the late flowering phenotype of *nit1/2/3*. The previous studies showed similar results, suggesting that the effect of MAF5 on flowering is very limited (Gu et al., 2013).

The FLC clade genes usually share expression regulators. The H3K4 methyltransferase COMPASS-like complex including the core components Ash2, RbBP5, and WDR5a deposits H3K4me3 and promotes the expression of *FLC, MAF4*, and *MAF5* (Jiang et al., 2011). The SWR1 chromatin remodeling complex promotes the substitution of H2A by H2A.Z (the histone variant promotes transcription) at *FLC, MAF4*, and *MAF5* chromatin, leading to increased *FLC, MAF4*, and *MAF5* expression (Cui et al., 2017). The FRIGIDA (FRI) is a key regulator of *FLC* expression. It recruits SWR1, COMPASS-like, and other chromatin modifiers to form a supercomplex to activate the expression of *FLC* (Li et al., 2008; Choi et al., 2011), and is possible to activate the expression of *MAF4* and *MAF5* (Kong et al., 2019). The flowering regulation by NIT1/2/3 depends specifically on MAF4 rather than FLC; therefore, these regulators mentioned here may not be involved in NIT1/2/3-mediated regulation of *MAF4*. Interestingly, we found the expression of *MAS*, a NAT–lncRNA, which is transcribed in the opposite direction of *MAF4* significantly increased in *nit1/2/3*. Also, *MAS* has been found to promote the transcription of *MAF4* through recruiting WDR5a, the core component of the COMPASS-like complex, to *MAF4* and enhance the H3K4me3 chromatin modification (Zhao et al., 2018). The high level of *MAS* and H3K4me3 in *nit1/2/3* suggested that NIT1/2/3 specifically regulate transcription of *MAF4* through manipulating *MAS*.

The molecular mechanism of MAFs inhibiting flowering has been studied. The study of Gu et al. shows that FLC, SVP, and MAFs may form several tetrameric complexes with different composition such as FLC-SVP-MAF3-MAF4 and SVP-FLM-MAF2-MAF4, to regulate flowering-time by directly binding to *FT* chromatin (Gu et al., 2013). They also suggested that the complexes made of MAFs and/or SVP without FLC may be the predominant ones available for floral repression in the rapid-cycling Arabidopsis accessions (e.g., Col). The direct interaction of MAF4 with FLC, FLM/MAF1, and MAF3 has been experimentally proved (Gu et al., 2013). Given that the DNA-binding domains in MAF4 are nearly identical with that of FLM/MAF1 and MAF3, which have been found to bind to *FT* chromatin (Searle et al., 2006; Li et al., 2008), MAF4 is predicted to bind to *FT* chromatin as well. Therefore, MAF4 may regulate flowering through directly binding to *FT* chromatin as a component of the MAF complex.

**Figure 9:**
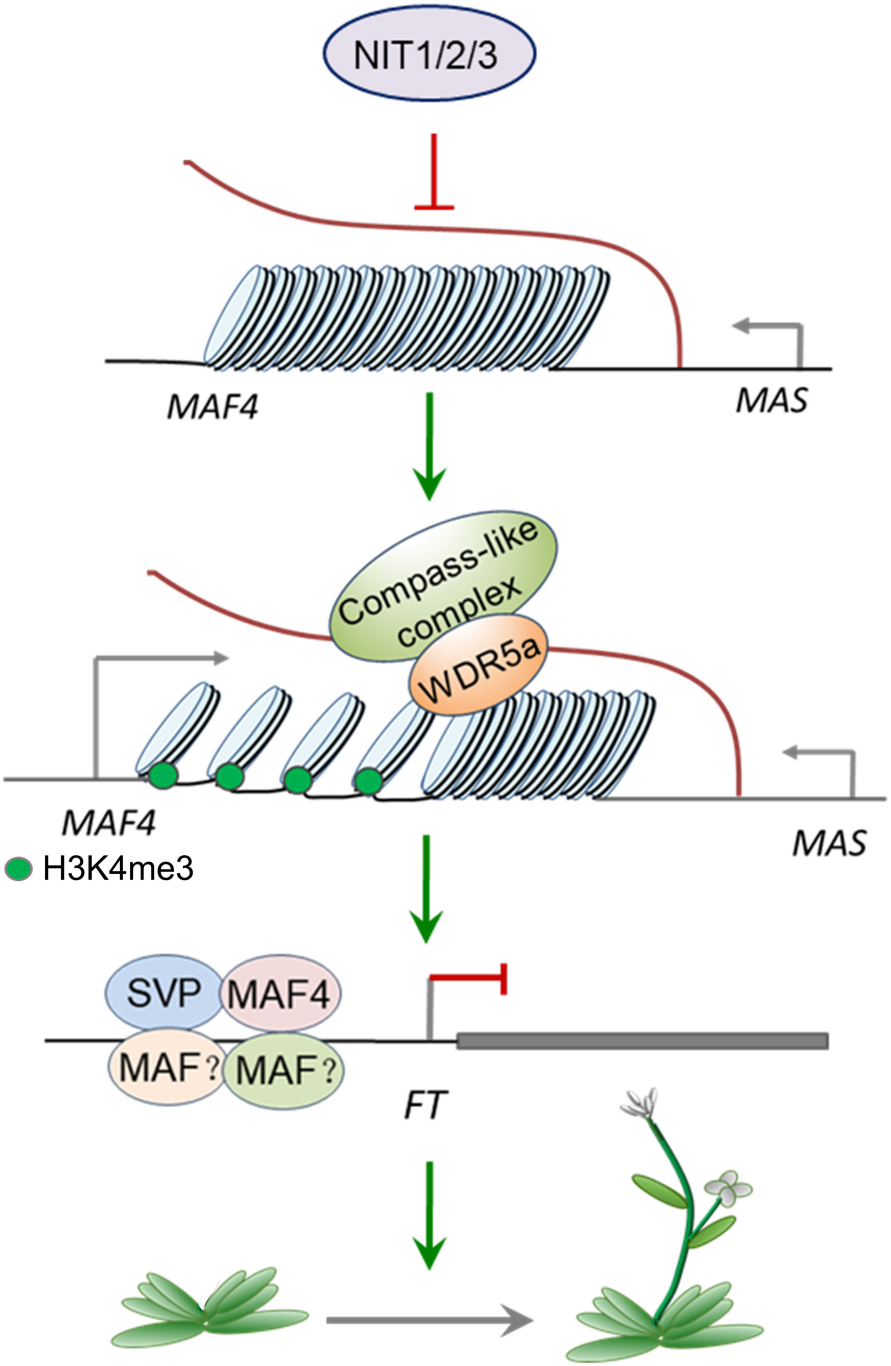
A model of NIT1/2/3 regulating flowering time.

Combined with the previous studies, we established a model of NIT1/2/3-mediated flowering-time regulation. As shown in Figure 9, NIT/1/2/3 inhibits the expression of *MAS*, a NAT–lncRNA transcribed in the opposite direction of *MAF4*. Low level *MAS* cannot bind enough WDR5a and fails to recruit COMPASS-like complex to *MAF4* chromatin, leading to the reduction of active H3K4me3 modification. The transcription of *MAF4* is then repressed, which reduces the formation of MAF tetrameric complex and leads to the transcription of *FT*.

Our study showed that nitrilases NIT1/2/3 positively regulate flowering by repressing the transcription of flowering inhibitor *MAF4*. However, there are still a lot of questions, such as “Is auxin really involved in flowering regulation?” “How is *MAS* regulated?” and “What are the functions of multiple alternative splice variants of *MAF4*?” need to be answered. In short, there is still a huge gap between our knowledge and the specific mechanism, which needs to be further explored.

## Data Availability Statement

The original contributions presented in the study are publicly available. This data can be found here: https://www.ncbi.nlm.nih.gov/sra/PRJNA826618.

## Author Contributions

JL and RL designed the experiment. SY conducted the experiment. TZ, ZW, and XZ participated in various parts of the experiment. JL and SY wrote the manuscript. All authors have read and approved the final manuscript.

## Funding

This work was supported by the National Natural Science Foundation of China (NSFC) (32070334).

## Conflict of Interest

The authors declare that the research was conducted in the absence of any commercial or financial relationships that could be construed as a potential conflict of interest.

## Publisher's Note

All claims expressed in this article are solely those of the authors and do not necessarily represent those of their affiliated organizations, or those of the publisher, the editors and the reviewers. Any product that may be evaluated in this article, or claim that may be made by its manufacturer, is not guaranteed or endorsed by the publisher.
