# Supplementary material for: Nitrilases NIT1/2/3 Positively Regulate Flowering by Inhibiting MAF4 Expression in Arabidopsis
Source: Front Plant Sci. 2022 May 17;13:889460. doi: 10.3389/fpls.2022.889460 (PMC9157433; doi:10.3389/fpls.2022.889460)
Supplement: Supplementary file 1 [file Data_Sheet_1.pdf]

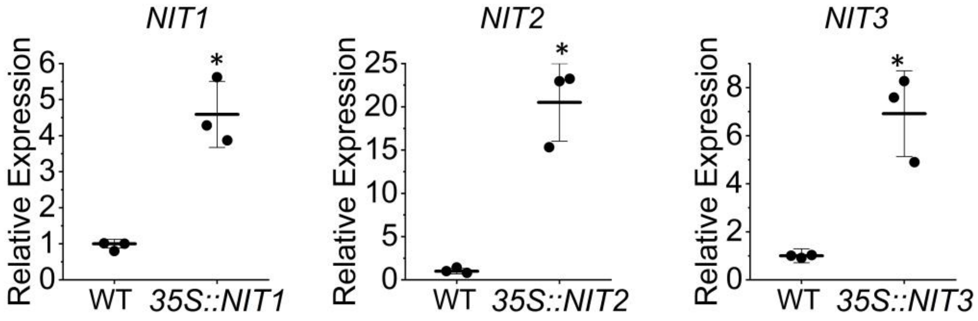

Figure S1. Relative expression levels of *NIT1/2/3* in transgenic lines. The error bars represent the standard error (three biological repeats). The asterisks at the top of the bar indicate significant differences between *35S::NIT1/2/3* and wild type (WT), $P < 0.05$(*) or $P < 0.01$(**) by Student's *t* test.

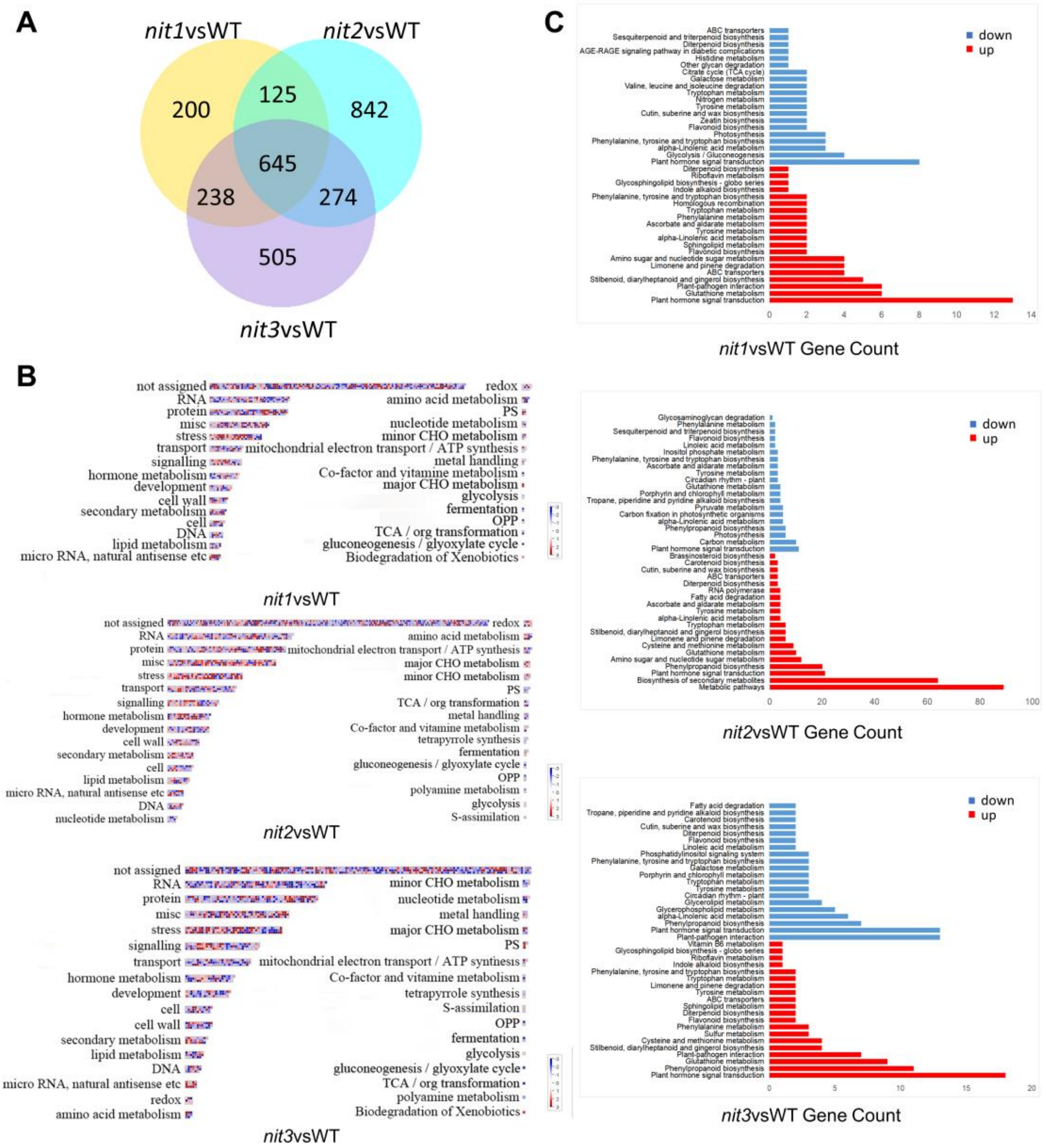

Figure S2. Transcriptome analysis of *nit1*/*nit2*/*nit3* in Arabidopsis. (A) Venn diagram showing overlapped differentially expressed genes (DEGs) between *nit1*, *nit2* and *nit3*. (B) GO enrichment analysis of the DEGs in *nit1*/*nit2*/*nit3* compared to wild type. Red squares represent up-regulated and blue squares represent down-regulated genes. (C) The top 40 (20 up-regulated, 20 down-regulated) enriched Kyoto Encyclopedia of Genes and Genomes (KEGG) pathway terms of DEGs.

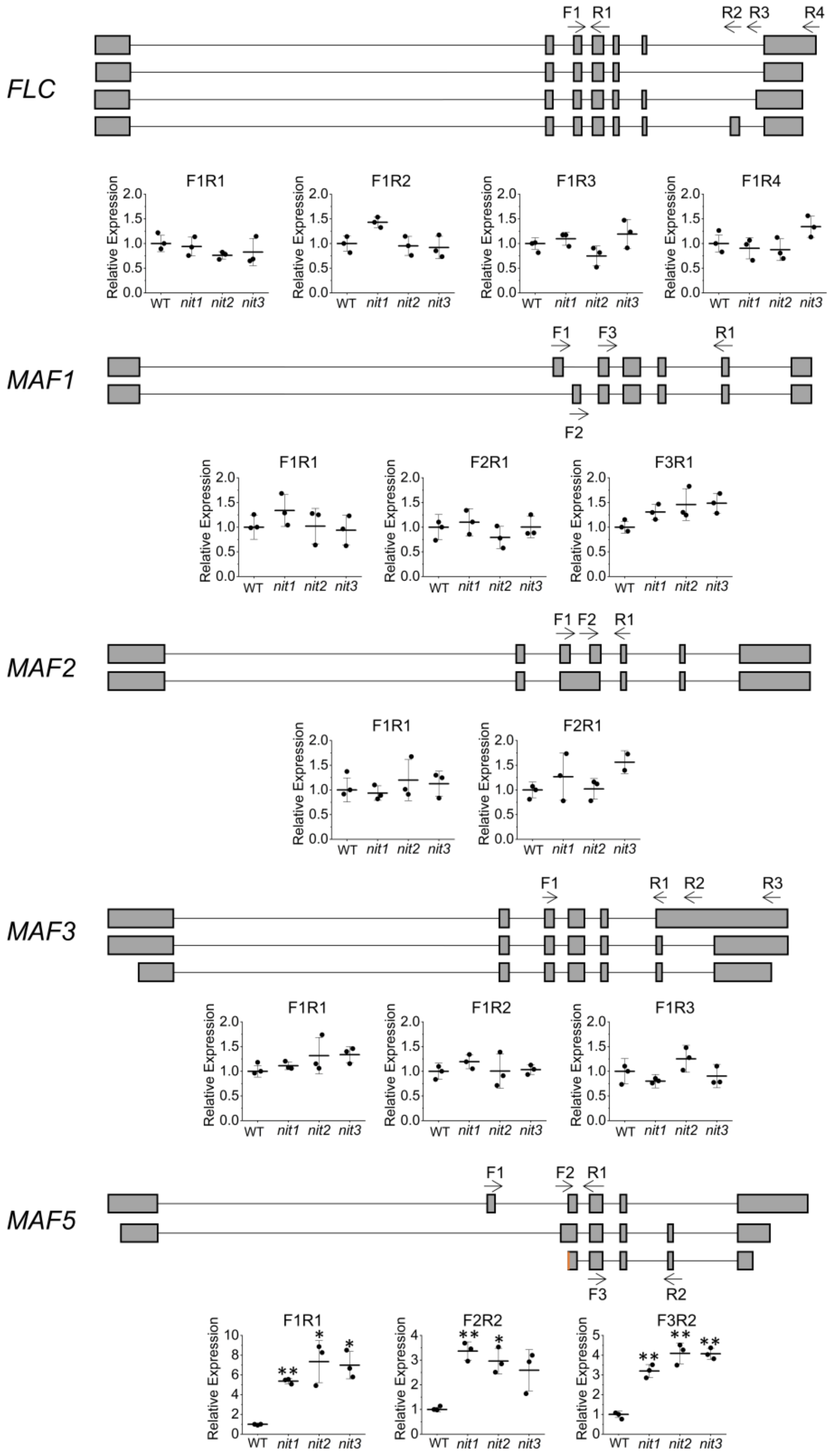

Figure S3. Relative expression levels of *FLC, MAF1, MAF2, MAF3* and *MAF5* splicing variants. Squares and lines represent exons and introns respectively. F means forward primer and R means reverse primer. Arrow indicates primer direction. The error bars represent the standard error (three biological repeats). The asterisks at the top of the bar indicate significant differences between *nit1/2/3* and wild type (WT), $P < 0.05$(*) or $P < 0.01$(**) by Student's t test.

Supplemental Table 1. Sequences of primers used in this study

Name	Sequence
NIT1-F	ATGTCTAGTACTAAAGATATGTCAACTGTC
NIT1-R	CTATTTGTTTGAGTCATCCTCAGC
NIT2-F	ATGTCAACTTCAGAAAACACTCC
NIT2-R	TTACTTGTTTGAGTCATCTTCCG
NIT3-F	ATGTCTAGTACTGAAGAAATGTCATCA
NIT3-R	CTATTTGTTTGATTCATCCTCTGC
NIT1p-F	AATCTTCTTTAGTTTTTATCTTAGGCT
NIT1p-R	CGAGTCTTTGTTTTTTACTTTGG
NIT2p-F	GATTGAGTCGACAGTAATACGAGAC
NIT2p-R	ACTTGAGCTTTAGTCTTACTTCGAT
NIT3p-F	AATGTATTTGGCTTGTCTTGTGTC
NIT3p-R	ACTCGAGTCTTTGTTTTTACTTTATAC
LBb1.3	ATTTTGCCGATTTCGGAAC
nit1-L	AAATAGGAGCCAAACTCACATTTC
nit1-R	CGCTAAACCGAACCTAAAACC
nit2-L	TGAGATGGTATACCACGGATG
nit2-R	AAACGGGTATCTTTCGGTTTG
nit3-L	GGTATTGCAGAACGAACC
nit3-R	GAGACTTGGGAGGCATT
maf4-L	TGATTACATATGCATGCGACC
maf4-R	TGAGTTATTGGGTCTCATGGG
ACTIN2-F	TTACCCGATGGGCAAGTC
ACTIN2-R	GCTCATACGGTCAGCGATAC
FKF1-F	GGAGGGTGTGGAAGACAAGG
FKF1-R	AGAAGGTGTCGCTGAGAAGC
TOC1-F	ATCCACATTCCCTTGAGCGG
TOC1-R	TTTGGTGCAGAGAAACCCGA
CDF3-F	TTGGTCTCGAAGCTCAGCAG
CDF3-R	ACAAGCCGTTGATCCGCTAA
CO-F	TCCCCCGTAGCTCGTCTGTGGTA
CO-R	GCGTGCTCCGGCTGCTTTTT
PHYA-F	TGATTCAGCCCTTCGGTTGT
PHYA-R	GACGCACTAGGAGCAGTGAA
FT-F	CTAGCAACCCTCACCTCCGA
FT-R	TCGTAACACACAATCTCATTGCCAAA
SOC1-F	GCTCATTGATGGACTCGTACATAAGTTGGT
SOC1-R	TTGACCAAACTTCGCTTTCA
AP1-F	CCAGAGGCATTATCTTGGGGAAGACTTG
AP1-R	GCTCATTGATGGACTCGTACATAAGTTGGT
LFY-F	TCATTTGCTACTCTCCGCCGCT
LFY-R	CATTTTTCGCCACGGTCTTTAG

FLC-F	TGTGGATAGCAAGCTTGTGG
FLC-R	TAGTCACGGAGAGGGCAGTC
MAF1-F1	ACATGCTGATGAACTTAGAGCCT
MAF1-F2	AGCCGGAGAAACCTCAATGT
MAF1-F3	CCACACAAGGAGTTACTAGAAACAGTCC
MAF1-R1	GCCAGAACCTGGTTCTCTTCTCTCAG
MAF2-F1	CTTGCAGAAAAAACTCGGAATTATCTGCCAC
MAF2-F2	TAGCACAAAGACACTTTTATCTCCCTC
MAF2-R1	CCTAGCTCTAGTTACGGACAGAGCAGTC
MAF3-F1	TCGGAATTATCTTCCACACAAGGAG
MAF3-R1	GCCAGAATCTGGTTCTCTTCTATCAGC
MAF3-R2	GCCTAGCTTCCATCAAACTCCT
MAF3-R3	CCAGAAACGTCTTCTTCCCCA
MAF4-F1	TTGTGAATCCTCCGTCGCTC
MAF4-F2	TCTTGATCTCTCCAGCATGGC
MAF4-F3	TGGCCAAGATCCTCAGTCGTTATGA
MAF4-F4	TCCAAGAGCAGTGTTTGGGT
MAF4-F5	GGAGCTTGTGAAGACCCATCA
MAF4-R1	TGGCCATGAAATCTACCGGG
MAF4-R2	TGCTGGTCTCTCTCCACAATG
MAF4-R3	TCTAGCAACTCCTTGTGCGAA
MAF4-R4	GCTGCTCTTCCAGGGACTTTAGACA
MAF4-R5	GCATAGCTAGGGATGGCGAA
MAF4-R6	TACTTGAGAAGCAGGAGAGTCTCC
MAF5-F1	TCCATGGAAGAGCAGCTCAA
MAF5-F2	GATGGAGCTTGTGAAGAACCTTCAGG
MAF5-F3	ACAAGCTGATGATCCTGAAACCT
MAF5-R1	CCACCTCGCTAGCTAGAACC
MAF5-R2	CAGCCGTTGATGATTGGTGGTTACTTG
MAF5-R3	ACAGACAGAGCACTCTTGAGC
MAS-F	GTGATGGTGGTTACTTGAGAAGCA
MAS-R	AACTGAATAAAGTTGTGATGGTG
MAF4-ChIP-F1	GGCAGAAAAAGACTTGTCGAGGCTGG
MAF4-ChIP-R1	GTGTGTTTTCCAAAAATCCAAAAATTTGAACCGAG
MAF4-ChIP-F2	CAAACGAATTGAGAACAAAAGCTCTCGACAAGT
MAF4-ChIP-R2	GCTGTAGAGTCTTCCGGTGGCAGA
MAF4-ChIP-F3	CAGTGGAAGAAAATAGCATGTGTACACATTGAGTAGC
MAF4-ChIP-R3	CGTACCAAATGTTTGTAGAGACTCAGTTTCTTTGG
MAF4-ChIP-F4	TTTCTTACAGATGGGGAAGATGAAGAAGTCTG
MAF4-ChIP-R4	GGTGATGGTGGTTACTTGAGAAGCAGGAG
MAF5-ChIP-F1	GCCCAGATTCGGCCCATTTA
MAF5-ChIP-R1	TGTCAATCGCGTACCCTGAA
MAF5-ChIP-F2	TCGGAAGAGTGAAGCCATGG
MAF5-ChIP-R2	ACGATGAAAAGAGCGACGGA

MAF5-ChIP-F3	GGGCCAAAAAGAGGTACCGA
MAF5-ChIP-R3	AGTGACAAGGACATCCCCAT

Supplemental Table 2. Proteins involved in regulation of *FLC* clade members

Gene	Locus	Function	Reference
FRI	AT4G00650	activates the transcription of *FLC*. FRI acts as a scaffold protein interacting with FRL1, FES1, SUF4, and FLX to form a transcription activator complex.	(Johanson et al., 2000; Choi et al., 2011)
FRL1<br>FRL2	AT5G16320<br>AT1G31814	family member of FRI-related genes that is required for the winter-annual habit.	(Michaels et al., 2004)
SUF4	AT1G30970	component of FRI complex.	(Kim et al., 2006)
FLX	AT2G30120	component of FRI complex, required for FRI-dependent activation of *FLC* expression.	(Andersson et al., 2008)
FES1	AT2G33835	component of FRI complex.	(Schmitz et al., 2005)
ABH1/ CBP20	AT2G13540	suppress *FLC* expression.	(Bezerra et al., 2004)
SWR1 complex		chromatin remodeling complex promotes the substitution of H2A by H2A.Z (the histone variant promotes transcription) at *FLC*, *MAF4*, and *MAF5* chromatin	(Cui et al., 2017)
SKIP	AT1G77180	activate the expression of *FLC* and *MAF4/5*.	
PIE1	AT3G33520	component of the SWR1 complex.	
ARP	AT2G41460	component of the SWR1 complex.	
SEF	AT5G37055	component of the SWR1 complex.	
FER	AT3G51550	positively regulates the transcription of *FLC/MAF*.	(Wang et al., 2020)
HEN4	AT5G64390	promotes transcription of *FLC* and *MAF4*.	(Ortuño-Miquel et al., 2019)
VOZ1<br>VOZ2	AT1G28520<br>AT2G42400	inhibit transcription of *FLC/MAF*.<br>inhibit transcription of *FLC/MAF*.	(Yasui and Kohchi, 2014)
FLY	AT1G33680	promotes transcription of *FLC* and *MAF*.	(Dai et al., 2020)
HUB1<br>HUB2<br>UBC1	AT2G44950<br>AT1G55250<br>AT1G14400	HUB1 and HUB2 interact with each other and with UBC1 and UBC2 to monoubiquitinate histone H2B and enhance H3K4 and H3K36	(Cao et al., 2008)

UBC2	AT2G02760	hypermethylation, increased *FLC/MAF* expression.	
HDA6	AT5G63110	binds to the chromatin of FLC/MAF4.	(Yu et al., 2011)
TOP1α	AT5G55300	binds to the genomic regions of *FLC*, *MAF4*, and *MAF5*, facilitating the expression of *FLC*, *MAF4*, and *MAF5*.	(Gong et al., 2017)
MLK4	AT3G13670	phosphorylates histone H3T3, repressing the transcription of *FLC/MAF*.	(Wang et al., 2021)
DEK3<br>DEK4	AT4G26630<br>AT5G55660	directly associate with chromatin of *FLC*, *MAF4* and *MAF5*, to promote their expression.	(Zong et al., 2021)
COMPASS-like		The H3K4 methyltransferase including the core components Ash2, RbBP5 and WDR5 deposits H3K4me3 and promotes the expression of *FLC*, *MAF4* and *MAF5*.	(Jiang et al., 2011)
Ash2R	AT1G51450	component of COMPASS-like complex.	
RbBP5	AT3G21060	component of COMPASS-like complex.	
WDR5	AT3G49660	component of COMPASS-like complex.	
